# Histopathological Determinants of Tumor Resistance: A Special Look to the Immunohistochemical Expression of Carbonic Anhydrase IX in Human Cancers

**DOI:** 10.2174/09298673113209990227

**Published:** 2014-05

**Authors:** G. Ilardi, N. Zambrano, F. Merolla, M. Siano, S. Varricchio, M. Vecchione, G. De Rosa, M. Mascolo, S. Staibano

**Affiliations:** 1 Department of Advanced Biomedical Sciences, Pathology Section Faculty of Medicine and Surgery, University of Naples "Federico II", address: via S. Pansini, n.5, Naples, Italy; 2 CEINGE Biotecnologie Avanzate SCaRL, Naples, Italy; Dipartimento di Medicina Molecolare e Biotecnologie Mediche, Universita􀀁 di Napoli Federico II, Italy, address: via S. Pansini n.5, Naples, Italy

**Keywords:** CA IX - Carbonic anhydrase, clinical-pathological features and biological outcome, hypoxia, immunohistochemistry, solid human cancers, stem cells markers, targeted therapy.

## Abstract

Intrinsic and acquired drug resistance of tumor cells still causes the failure of treatment regimens in advanced human cancers. It may be driven by intrinsic tumor cells features, or may also arise from micro environmental influences. Hypoxia is a microenvironment feature associated with the aggressiveness and metastasizing ability of human solid cancers. Hypoxic cancer cells overexpress Carbonic Anhydrase IX (CA IX). CA IX ensures a favorable tumor intracellular pH, while contributing to stromal acidosis, which facilitates tumor invasion and metastasis. The overexpression of CA IX is considered an epiphenomenon of the presence of hypoxic, aggressive tumor cells. Recently, a relationship between CA IX overexpression and the cancer stem cells (CSCs) population has been hypothesized. CSCs are strictly regulated by tumor hypoxia and drive a major non-mutational mechanism of cancer drug-resistance. We reviewed the current data concerning the role of CA IX overexpression in human malignancies, extending such information to the expression of the stem cells markers CD44 and nestin in solid cancers, to explore their relationship with the biological behavior of tumors. CA IX is heavily expressed in advanced tumors. A positive trend of correlation between CA IX overexpression, tumor stage/grade and poor outcome emerged. Moreover, stromal CA IX expression was associated with adverse events occurrence, maybe signaling the direct action of CA IX in directing the mesenchymal changes that favor tumor invasion; in addition, membranous/cytoplasmic co-overexpression of CA IX and stem cells markers were found in several aggressive tumors. This suggests that CA IX targeting could indirectly deplete CSCs and counteract resistance of solid cancers in the clinical setting.

## TUMOR STROMA, CANCER HETEROGENEITY, AND DRUG-RESISTANCE

More than a decade after the completion of Human Genome Project, an unprecedented flow of genomic and epigenetic information, paralleled by the rapid advancement of technology and drug discovery, has opened up new avenues to improve the outcomes of cancer patients. New promising biomarkers and molecularly targeted anti-tumor drugs are increasingly becoming available.

This is a particularly encouraging scenario considering that epidemiologists have predicted a progressive boost of new cancer cases worldwide, up to an extent exceeding 22 million by 2030 [1, 2].

However, cancer is a heterogeneous disease, both from a genetic and epigenetic point-of-view, representing the major hurdle that limits the actual success of most of the new therapeutic strategies. The cells composing a clinically appreciable malignant tumor, in fact, may contain specific sets of mutations in different sub-clones, most of which absent from the founder neoplastic population [3-5].

Recently, it has been reported that the cellular subpopulations that characterize a cancer may trans-differentiate into another [6-8]. This acquired heterogeneity favors the evolutionary adaptation of cancer cells to adverse environmental conditions, either linked to specific intra-tumor characteristics or induced by chemotherapy, leading to disease progression and eventually death of patients with advanced and metastatic disease [4].

Multidrug resistance is a complex and dynamic phenomenon [9], variously encompassing the increase of drug detoxification mechanisms, changes in drug kinetics, the expression of multidrug efflux pumps (i.e., MDR-1/PgP/ATP-binding cassette family of drug transporters and cytochrome P450 family of enzymes) [10, 11], alteration of apoptosis [12, 13], amplification of drug targets (i.e. gene amplification of receptors targeted by tyrosine kinase inhibitors [14].

A growing number of evidences also indicated that changes in tumor microenvironment may contribute to resistance to both chemotherapy and radiotherapy. In particular, the importance of stroma-mediated chemosensitivity has been recognized and is the basis for the development of new anticancer agents [14, 15].

Stromal components can constitute greater than 50% of tumor mass [16]. Malignant tumors have the capacity to mobilize the host normal tissues to support and protect them, evolving a desmoplastic stroma, which supports tumor growth and invasion through the expression of growth factors and cytokines in the extracellular space [17] and tumor associated paracrine stimuli further promoting mesenchymal cell growth and survival [15].

When solid cancers outgrow, their vasculature becomes inadequate [18, 19] and glucose metabolism via glycolysis becomes dominant [20, 21].

## TUMOR HYPOXIA, CA IX AND CANCER STEM CELLS

Hypoxia has emerged as a major tumor microenvironment feature linked with a more aggressive malignant phenotype. The frenetic proliferation of cancers is accompanied by aberrant signaling producing poor quality vasculature and, over the half of solid malignant tumors, harbor multiple regions of hypoxia heterogeneously distributed throughout tumor masses [22, 23].

The activation of the hypoxia-inducible factor 1 and 2 (HIF-1/2) [24], and GLUT-1 [15] represents the immediate response of cancer cells to hypoxia [16].

In addition, as an early response to hypoxia, the over-expression of carbonic anhydrase IX (CA IX) on the surface of hypoxic tumor cells takes place too. Originally identified in HeLa cells [25], CA IX is a HIF-1a regulated, trans membrane, dimeric protein [26] belonging to a large family of zinc metallic-enzymes that catalyze the reversible hydration of carbon dioxide (CO_2_) to bicarbonate and protons (CO_2_ + H_2_O ↔ HCO_3_^-^ + H^+^) [27].

Afterwards, CA IX act as a “catalytic converter” which [28] shuttles bicarbonate into malignant cells cytoplasm, protecting the cytosol from acidic pH levels [29, 30], while forcing protons to concentrate in the extracellular space further contributing to the acidic extracellular microenvironment. 

CA IX action results in neighboring normal cells’ death and accelerates extracellular matrix’s degradation [31, 32], favoring survival, proliferation [33], protease activation, growth factor production [34-40], invasion and metastasizing ability of acid-resistant cancer cells [28, 34, 35]. Genetic silencing of CA IX in preclinical tumor models *in vivo* has demonstrated the requirement of CA IX for the growth of hypoxic tumors and their metastasis [41].

Besides its activity in inducing the acidification of tumor extracellular pH, CA IX also physically perturbs intercellular contacts through competition with E-cadherin/β-catenin [42-44].

Furthermore, CA IX may favor the acquisition of stemness phenotype by hypoxic cancer cells, modulating E-cadherin-mediated cell adhesion and leading to epithelial-mesenchymal transition (EMT) [44]. These findings indicate that the action of CA IX in hypoxic tumors extends further beyond the control of intra-tumor pH.

**Fig. (1):**
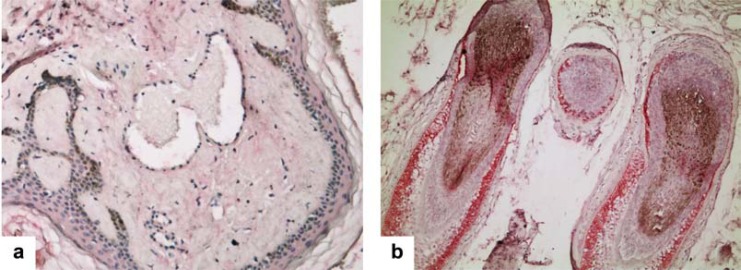
**a**) Human skin: absence of CA IX staining in normal epidermis, dermis, and endothelium of a dermal angioma; **b**) High level-CA IX immunohistochemical expression in the infundibulum and outer sheath of hair follicles of human skin.

Transient CA IX expression is observed in the relatively hypoxic fetal environment, where it is restricted to immature tissues of mesodermal origin, skin and ependymal cells [45]. In normoxic adult human tissue, Ca IX results expressed at low level in the basolateral surface of gastric mucosal epithelium [46] and in the proliferating crypts of the duodenum, jejunem and ileal mucosa [47], mesothelium [48] remnants of the coelomic epithelium (i.e., rete ovarii and rete testis) [48, 49], gallbladder, focally in pancreatic acini [50], while at high level in the infundibulum and in the outer sheath of hair follicles and the sebaceous units of the skin [45, 48, 51] (Fig. **1 a, b**).

Overall, in normal adult tissues, CA IX expression seems to be related to the cell origin and functional status [45]. As an example, increased levels of CA IX expression in the basal cells of squamous or respiratory epithelia may be found at the sites of tissue repair due to injury or inflammation [45].

The inconspicuous presence of CA IX in normal adult tissue justifies the observation that an interference with its function in physiological conditions doesn’t seem to produce relevant consequences.

By converse, CA IX is ectopically overexpressed by a considerable variety of frequently occurring solid tumors without an obvious correlation with specific tumor histotypes and it has been frequently reported associated with the occurrence of metastases, shorter disease-free and/or disease specific survival of patients [25, 52-80].

Pharmacologic interference of CA IX catalytic activity using monoclonal antibodies or CA IX-specific small molecule inhibitors has been shown, recently, to impair primary tumor growth and metastasis. As an example, the effects of radiation and chemotherapy were strongly augmented after CA IX interference and were accompanied by a higher rate of apoptotic cell death in glioblastoma [81].

Even more interestingly, several protein tyrosine kinase inhibitors (PTKIs) in clinical use (i.e. imatinib and nilotinib) were recently shown to be nanomolar CA IX inhibitors [82, 83]. This finding explains that the potent antitumor effects of these molecules in several types of malignancies may be due also to the inhibition of CA IX, besides to the PTK inhibition [84].

Notwithstanding these exciting findings, the actual predictive value of CA IX in the identification of drug-resistant cancers remains still uncertain. The rationale of this disappointing phenomenon resides mostly on the basis that multiple interacting mechanisms usually drive the emergence and development of tumor drug resistance.

Cancer cell plasticity constitutes an emerging feature, which frequently underlies drug resistance. It refers to the ability of a cell to reversibly change lineage and modify cell behavior, mostly as a consequence of any changes in the adjacent tumor environment [14].

The best-described example of cancer cell plasticity is represented by the epithelial to mesenchymal transition (EMT) and the reverse of this process; the mesenchymal to epithelial transition (MET) [85]. A causal link has been demonstrated between the EMT and the acquisition of stem-like activities and chemo-resistance [85-93].

Recently, it has been evidenced the possible relationship between CA IX overexpression and cancer stem cells (CSCs), which typically reside in hypoxic cell niches.

Hypoxia is a major environmental condition able to induce profound effects on cancer stem cells [15]. It influences the activation of differentiation pathways, stem cell identity maintenance and the metastatic potential of CSCs. The highly tumorigenic fraction of side population (SP) cells/tumor stem cells classically resides to hypoxic areas of solid tumors, which behave as ideal niches for these acid-resistant cells [94].

The loss of transformed cells’ phenotype in response to hypoxia and hypoxia-induced CA IX is similar to that associated with epithelial-mesenchymal transition (EMT), both in embryonic development and in metastasis [95], and has been found to be attributed, at least in part, to CA IX-induced interference with the Rho/ROCK (Rho-associated kinase) signaling pathway affecting β-catenin [44].

H-J Shin and colleagues have, recently, evidenced the direct binding of CA IX with DKK1, a secreted transcriptional target along with vimentin and fibroblast growth factor 20 (FGF20) in the Wnt/β-catenin/TCF signaling pathway [96-99].

Moreover, critical pathways downstream of CA IX, as the mTORC1 axis, Notch1 and Jagged1, are among the major regulators of cancer stem cell function and drivers of stemness [100].

These evident cross talks between CA IX, CSCs phenotype and EMT are perfectly in line with the idea introduced by Liao SY *et al*., in 2009, that CA IX may be a marker for stem cells in certain tissues, based on the localization of CA IX expression in sites normally corresponding to stem cell niches (e.g.in rare cells or niches in late stages of fetal development, and postnatal harboring adult stem cells, as the hair follicles, including the bulge, sebaceous gland, outer root sheath and infundibulum, plus rare cells in the inter-follicular zone [101, 102], or Müllerian-type columnar and reserve cells of the cervix [45, 103], and in the small and large intestine stem cell niches).

Very interestingly, it has been reported that, besides cell origin and differentiation, ion transport (i.e., low extracellular pH in normal digestive tract), and cellular hypoxic condition, CA IX expression may also serve as a biomarker for transit amplifying cells [45].

This emerging inter-relationship with the cancer stem cells population supports the idea that targeting CA IX in hypoxic advanced tumors may exert pleiotropic beneficial effects extending also to cancer stem cells, without affecting normal tissues. The recent finding that inhibition of CA IX expression with small-molecule inhibitors in breast cancer cell lines, in primary metastatic breast cancer cells and in mice bearing orthotopic breast tumors, results in the inhibition of breast CSC expansion in hypoxia, further supports this hypothesis [100].

## IMMUNOHISTOCHEMICAL EXPRESSION OF CA IX IN HUMAN SOLID CANCERS

### Co-expression of CA IX and Stem Cells Markers

In line with these concepts, we observed a strict association between CA IX overexpression and the stem cell markers, CD44 and nestin, in several aggressive, metastasizing cancers, within a series of 150 human solid cancers with different histogenesis.

**Fig. (2):**
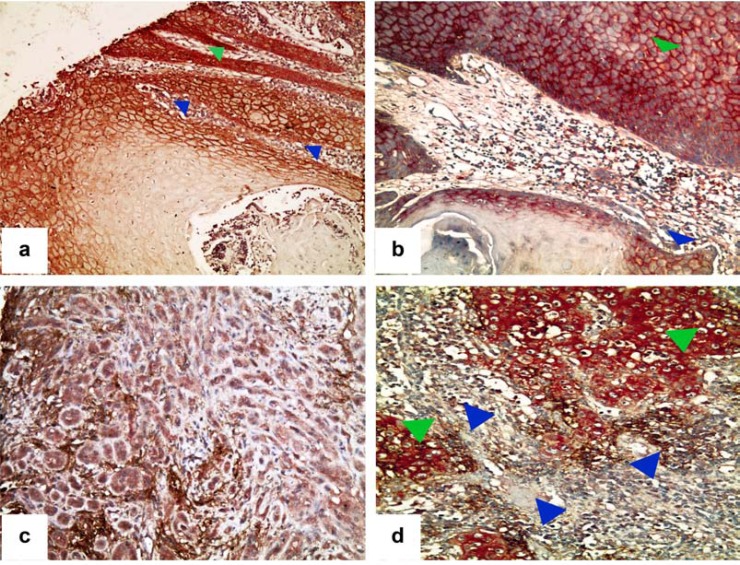
CA IX immunohistochemical expression in tongue squamous cell invasive cancer: **a**) A strong cytoplasmic membrane CA IX immunostaining is appreciable in squamous cell cancer cells lining a central necrotic area, in an advanced tongue cancer (blue arrowheads) and in peripheral infiltrating tumor sheets (green arrowheads) **b**) A strong co-expression of CA IX and nestin characterizes both internal areas (green arrowhead) and the invasive front (blue arrowhead) of a case of tongue squamous cell invasive cancer with poor prognosis. **c**) A case of tongue squamous cell invasive cancer showing CA IX expression in the extracellular space (tumor stroma). **d**) A case of tongue squamous cell invasive cancer with poor prognosis, showing a strong expression of the stem-cells marker CD44 in tumor cells (green arrowheads) the expression of CA IX, mostly in the extracellular space (tumor stroma, blue arrowheads).

**Fig. (3):**
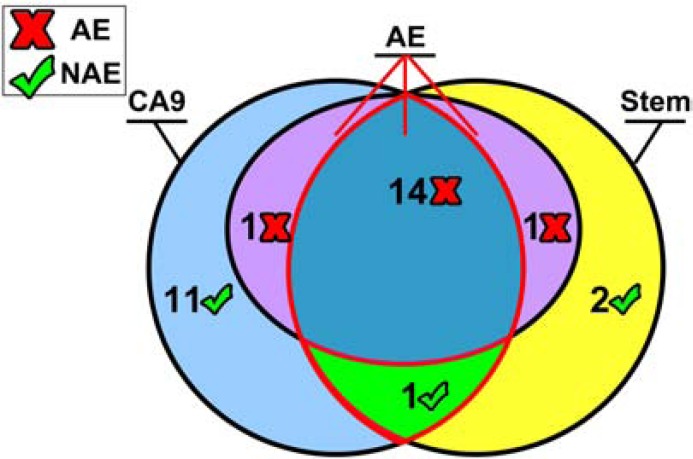
Three-set Venn diagram showing: CA IX positive cases (tot 27: light blu circle), Stemness markers positive cases (tot 18: yellow circle), Adverse events occurrence during follow-up (tot 15: pink ellipse); the dark blue region shows cases with an adverse event co-expressing CA IX and Stemness markers (14); the green region shows 1 case both expressing CA IX and stemness markers with no adverse events occurrence at follow-up. The red X indicates the adverse events. The green flag indicates no adverse event occurrence. Numbers of cases are shown in each region.

This association was particularly striking in a series of tongue cancers selected from a previous collection tested for CAF1/p60, sorting them by grading, staging and biological behavior, in order to represent the various prognostic categories [104]. The series was composed of 30 cases (11 well differentiated/G1, 14 moderately differentiated/G2 and 5 poorly differentiated/G3; 18 M; age range: 48-95 years; follow-up time 15 - 88 months, during which 2 patients developed relapses and distant metastases, 1 distant metastases and died for disease and 14 local relapse, distant metastases and died for disease). The association of CA IX overexpression and stem cells markers was highly significant (p<0.0001), constituting “the” hallmark of almost all the tumors with poor prognosis (Fig. **2 a, b**; Fig. **3**). This finding turns out to be extremely interesting, considering that tongue cancer is the most common type of squamous cell carcinoma of all the head and neck region, with an endless increase of annual incidence. As a rule, it rapidly progresses and frequently metastasizes, showing a poorer prognosis among the cancers of the oral cavity [101]. Chemotherapy often represents the only therapeutic chance for tongue cancers detected at a late stage, but frequently these tumors develop multidrug resistance.

### Stromal Expression of CA IX

Several cases of advanced tongue cancers were characterized by a prevalent stromal localization of CA IX immune-staining (Fig. **2 c, d**), which did reach statistical significance for that concerning the advanced tumor stage (P=0.007) and the occurrence of adverse events during the follow-up (P=0.002).

**Fig. (4):**
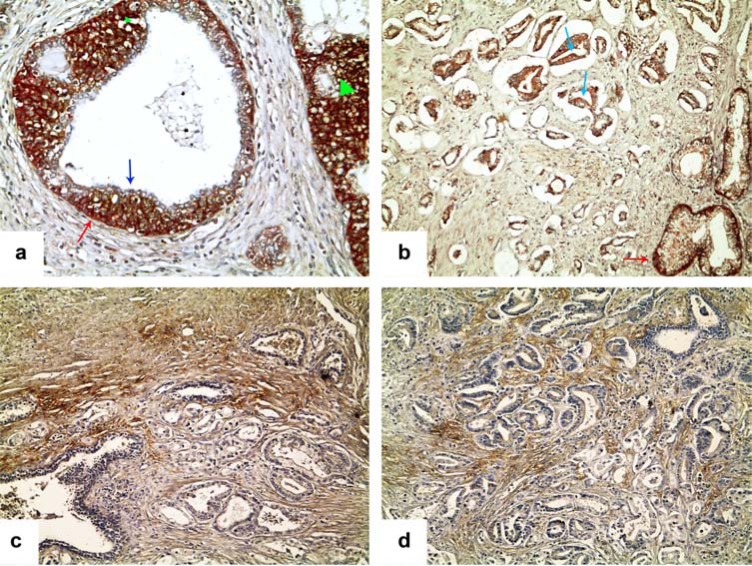
CA IX immunohistochemical expression in human prostate gland: **a**) CA IX epithelial expression in benign hyperplastic prostate glands, in areas of basal cell hyperplasia (green arrowheads); The staining for CD44 was restricted to the basal glandular epithelial layer (red arrow); the differentiated luminal epithelial cells were negative for both the markers (blue arrow). **b**) A case of prostate adenocarcinoma, showing definite epithelial expression of CA IX in neoplastic glands, with frequent membrane signal reinforcement (blue arrows). CD44 was expressed focally at the basal level of non-neoplastic prostate glands (red arrow). **c**, **d**) Two cases of prostate adenocarcinoma, showing only a strong extracellular signal for CA IX mainly located at the invasive tumor front.

A few cases of prostate cancers analyzed also showed a stromal localization of CA IX immune-reactivity, as well. We tested 30 prostate adenocarcinomas for CA IX expression, selected from a wider series previously tested for BAG3 expression [105], upon stratification into three groups, according to the combined Gleason score: low-grade (n=10), intermediate-grade (n=12) and high-grade carcinomas (n=8). The mean age of patients was 65.7 years; the follow-up time ranged between 30 and 64 months, during which 3 patients developed distant metastases (at surgery time), 2 developed distant metastasis and died for the disease. Prostate adenocarcinomas resulted, frequently, non-expressing CA IX, according to what previously reported by Donato DP and coll. [106-108]. However, in several cases, we found either an epithelial expression of the protein in neoplastic glands, with a frequent membrane signal reinforcement, (Fig. **4b**) and a definite immune-reactivity of the tumor stromal (Fig. **4c, 4d**). In 5 samples (2 cases with Gleason’s score 7 and 3 with Gleason’s score 8), 3 of which were classified as stage III and 2 as stage IV, the stromal signal was significantly correlated with adverse events occurrence during follow-up (p=0.0005); in fact 2 out of 5 developed metastasis, and 2 out of 5 developed metastasis and died of disease. 

Interestingly, we found also a strong CA IX epithelial expression in several prostate benign hyperplastic glands, in the areas of basal cell hyperplasia (Fig. **4a**), whereas the existing data of the literature report that non-neoplastic prostate glands are negative for said protein. We are not able to correctly understand this finding at present; however, it could be related with the hyper-proliferative status of benign prostate cells, instead of their hypoxic state. A proper interpretation of these data will, probably, arise from further studies on larger series of cases.

Several reports have signaled the occurrence of stromal expression of CA IX in solid malignant tumors while, as a rule, the only mesenchymal tissues that retained CA IX expression, in normal human tissues, are the submesothelial stromal cells, the meniscus and the nucleus pulposus of the vertebrae [45].

In cancers, stromal CA IX expression has been variously correlated either with worse survival chances [58, 109-111] or with a better outcome of patients [112].

In a study on Head and Neck Squamous Cell Carcinomas (HNSCC), N. Brockton and colleagues reported that a high stromal CA IX expression was associated with significantly reduced overall survival in patients with HPV-negative cancers, suggesting that CA IX expression in these tumors could identify patients with poor prognosis and inform therapeutic strategies [113]. Several other studies reported, instead, a significant relationship with HNSCC cells overexpression of CA IX, advanced stages of cancer [114] and resistance to chemo-radiotherapy [69, 71, 75, 78, 114-118].

Moreover, CHRISTINA S. KONG and colleagues found no relationship between HPV status, tumor pO2 and tumor (epithelial) CA IX staining, suggesting either that HPV infection does not influence tumor hypoxia, nor correlates with EGFR expression [119]. Moreover, Eriksen *et al.* did not find a correlation between CA IX expression and head and neck cancers’ prognosis [120].

It has to be outlined that the scenario is further complicated by the well-known inter- and intra-tumor heterogeneity of HNSCC, that significantly accounts for their particularly poor prognosis [121, 122], with a relative 5-year survival rate of patients (which has not significantly changed [123], besides the progress of diagnosis and therapy registered during the last decade).

Nevertheless, most of the existing reports have evaluated the expression of CA IX in HNSCC as in the other solid tumors “within the tumor cells”, neglecting its presence in tumor-associated stromal. This could be the cause of at least a partial misinterpretation of the expression data.

According to that, it has been recently hypothesized [67, 124] that the poorer survival associated with high stromal CA IX expression may be attributable, rather than hypoxia per se, to the acidification of the tumor microenvironment [125], which confers to cancer cells a survival advantage and contributes to the invasiveness and poor prognosis, activating proteases and disrupting cell adhesion molecule function [31, 43, 126].

Mounting evidences confirm that the transcription of many genes related to cell adhesion and cytoskeletal organization, can be induced by the overexpression of CA IX [44]. Dramatic changes of actin filaments and focal adhesion complex proteins, similar to the pattern of HeLa cells exposed to a concentration of 0.1% oxygen, have been documented in CA IX-transfected cells, indicating that the CA IX-induced changes take a part in the gain of increased metastatic potential of cancer cells under a hypoxic microenvironment [44].

Pericellular acidosis scattered around tumor cells, leads to the “redistribution” of the cell surface and an increased secretion of active cathepsin B [127-129], a lysosomal cysteine protease involved in degrading processes associated with tumor invasion [127]. In addition, the CA IX-induced acidic extracellular pH can influence the uptake of anticancer drugs and modulate the response of tumor cells to conventional therapy [36-40, 130].

### Expression of CA IX in Tumor Cells

Overall, the analysis of the dataset in the available literature shows a striking association between the presence of a solid malignant tumor and CA IX expression. 

**Fig. (5):**
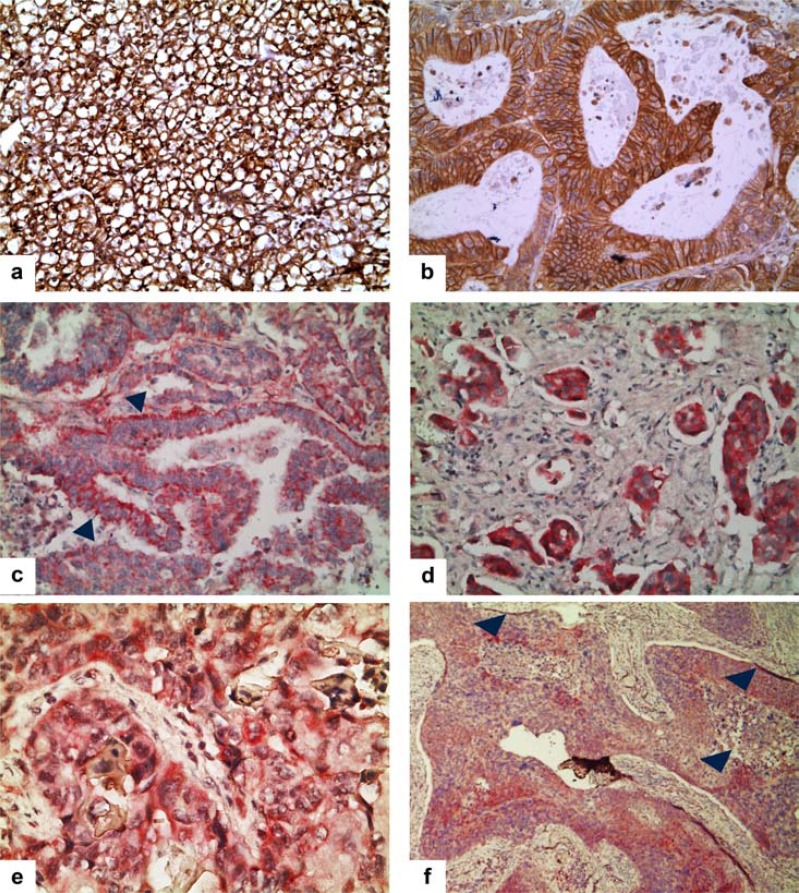
**a**) Strong cytoplasmic membrane CA IX expression in renal cell carcinoma. **b**) Strong membranous expression of CA IX in colon cancer. **c**) Definite cytoplasmic membrane CA IX immune-staining in endometrial carcinoma. **d**) Strong membranous expression of CA IX in high-grade, invasive bladder cancer with squamous differentiation. **e**) Highly variable CA IX immune-staining of neoplastic cells in gastric cancer of intestinal histotype. **f**) A case of squamous cell invasive cancer of the uterine cervix: an appreciable staining for CA IX was restricted mainly to the peri-necrotic, hypoxic areas of tumor (blue arrowheads).

According with previous data of the literature, we found the strongest cytoplasmic membrane expression in Renal Cell Carcinoma [48, 131-133] (Fig. **5a**).

However, the significance of CA IX expression in this type of cancer is quite unique, among malignant tumors. RCCC is, in fact, a VHL “disease” [132] in which hypoxia-independent CA IX regulatory pathways may occur. In RCCC, mutation of the VHL tumor suppressor gene stabilizes, indeed, HIF-1alpha both in normoxia and hypoxia, driving the hypoxia-independent, high expression of CA IX in 95–100% of cases. 

For this reason, [134] CA IX is considered a diagnostic marker for RCCC. Very interestingly [133], this overexpression has been found to be associated with an overall good prognosis [48], with longer disease-specific survival of RCCC patients with metastatic disease, or improved recurrence-free survival in the case of localized RCCC. It has been hypothesized that CA IX can function, in these tumors, as a chaperone with immune-adjuvant properties [134]. This could constitute the rationale for the good response of patients to treatment with interleukin (IL)-2 [106].

It is also believed that the high expression of CA IX in RCCC tumors may be the result of a positive feedback loop induced by epidermal growth factor receptor (EGFR), which is frequently overexpressed in RCCC. According to recent results, EGFR phosphorylates CA IX in its intracellular domain at a tyrosine side (CA IX-pY). CA IX-pY activates Akt which promotes the expression of Hif-1a which, in turn, enhances the expression of CA IX facilitating acidosis and tumor cell invasion [135].

A strong membranous expression of CA IX was also found in the majority of the most frequent solid human cancers. Contrary to RCCC, however, the high levels of CA IX are, almost always, associated with advanced stage and/or high grade of tumors and, often, with their poor prognosis (Table **1**). This was the case for our series of 11 cases of high-grade, invasive bladder cancers that, according to previous reports, were found extensively immune-reactive for the protein [73, 106, 110, 136] (Fig. **5d**).

As well, according to literature [110], CA IX overexpression correlated with tumor stage (P 0.045) and nodal metastases (P<0.001), in our series of colon cancers.

A strong cytoplasmic/membrane overexpression of CA IX was also observed in our study population (Fig. **5b**), consisting of 30 adenocarcinomas of the colon (10 F; 20 M; age ranging from 43 to 86 years; 17 cases were well differentiated (G1, 5 moderately differentiated/G2 and the remaining 8 cases poorly differentiated/G3; follow up time 7-62 months, during which one patient developed a distant metastasis; at surgery time, 8 cases were at advanced stage), selected from archive files of the Department of Advanced Biomedical Sciences of the University “Federico II” of Naples. 

A definite cytoplasmic/membrane signal was also found in 5 cases of endometrial carcinomas (Fig. **5c**).

In gastric cancers, CA IX has been reported expressed with variable degrees, being prevalently detectable in intestinal-type of cancers, mainly at the invasion front [137], as the result of hypo-methylation in the CA IX gene promoter [138, 139]. We selected, from archive files, 30 cases of gastric adenocarcinoma, basing on grading, staging and clinical outcome (13 Females; 17 M; age range 36-88 years; 1 at stage I, 10 at stage II, 12 at stage III and 7 at stage IV). We found a highly variable immune staining of neoplastic cells only in the intestinal-histotype, (Fig. **5e**), without significant correlation with the clinical and pathological features.

The existing studies exploring the correlation between CA IX expression in primary Squamous Cervical Cancers and patients’ outcome have given contrasting results. Most reports suggested that CA IX expressing-clones in primary tumors are highly metastatic [61, 62], since the primary tumors that showed high CA IX expression also showed high CA IX expression in the matching metastatic lymphnodes [62].

By converse, another study did not find statistical significance between CA IX expression in primary cervical tumors and clinical and pathological features of worse prognosis, including tumor stage and histological subtype [76].

We selected 19 archival cases of cervical cancers, representative of different prognostic group, taking into account lesion’s grading, staging and follow-up data (age range 24-83 years; 3 well differentiated/G1, 4 moderately differentiated/G2, 12 poorly differentiated/G3;9 at stage I, 1 at stage II and 9 at stage III). We found that an appreciable staining for CA IX was restricted mainly to the peri-necrotic, hypoxic areas of tumors (Fig. **5f**), with a significant correlation with histologic grade and stage (p<0.005).

### CA IX Expression and the “Meta-heterogeneity” of Human Cancers

The heterogeneity of results concerning the CA IX expression between different studies in human solid cancers may have several possible origins.

It is actually thought that it is mainly linked to the statistical studies’ lack of power and by the use of antibodies that show different sensitivities and specificities [58].

In addition, it may be also due to sampling error in the presence of high intra-tumor heterogeneity [76], or it may be correlated with the topographical localization of CA IX, in tumors. This topic deserves further attention.

As an example, it has been recently reported that, at least in selected cancer types, CA IX may undergo stabilization, internalization and nuclear redistribution following hypoxic stimulation [140]. Additional reports of nuclear/perinuclear CA IX staining in human tumor tissues described its association with poor prognosis [52, 141].

These findings suggest that CA IX could have a nuclear function in signaling and transcription, similarly to other imported trans-membrane proteins, such as CD44 and EGFR; however, its nuclear functions through protein−protein interactions rather than through direct binding to DNA doesn’t be ruled off, at present.

Definitive experimental evidence for the CA IX role in nucleus is still missing, but it seems likely that future therapies should take into account the molecular targeting of these different intracellular subpopulations of CA IX [140].

In addition, only a part of the existing studies, performed the analysis of nodal stages and tumor grades with respect to high or low levels of CA IX, therefore, in several instances, any meta-analysis may be performed [118].

Finally, in line with what previously discussed, CA IX expression may differ depending on the variable presence of CSCs in each case of tumor.

Overall, according to that observed by Horswell *et al*., this heterogeneity of heterogeneity (“meta-heterogeneity”) is to be carefully considered when determining diagnostic and prognostic analysis of a solid cancer, particularly for what it concerns any kind of drug resistance’s prevision [142]. This is an area of active research as, to date, patient response to most of the traditional and new classes of ‘targeted’ drugs still varies from profound curative responses [143, 144] to transient [145, 146] or poor responses [147], without any real possibility to predict it. 

Several cases of human cancers, according to the general branched evolutionary model [148], may develop and retain multiple subclonal populations from their onset, with the existence of resistant subclones even at treatment initiation. A dominant clone may be susceptible to the initial treatment, whereas a resistant subclone could expand as the drug destroys the initially dominant clone.

Up to date, we need more efficient tools to evaluate the real situation of each single case of malignant tumor, before starting treatment, and even to properly monitor its evolution during traditional chemotherapy or new target therapies. CA IX looks very promising, at this regard [149-151].

CA IX-specific therapeutic modalities in cancer treatment are: monoclonal antibodies directed at the PG-like domain, as cG250 [152], or targeted against its catalytic domain [153-155], small molecule inhibitors [27, 29], and compounds based on sulfonamide/sulfamates and coumarins [156, 157].

The trans-membrane location of CA IX will be extremely useful for cell sorting analyses, and its overexpression can confidently be appreciated by immunohistochemical analysis on routine tissue sections [45]. It may also provide an extremely promising tool for therapeutic regimens that target CA IX-expressing cancer stem cells for destruction, without compromising normal tissues [158].

## CONCLUSIVE REMARKS

The massive next-generation sequencing of whole exomes and genomes is discovering genes potentially involved in resistance to therapy of human cancers, and automated large-scale screening of cancer drugs continues to identify promising new combinatorial regimens, on the basis of new biological hypotheses [159].

The majority of the existing data indicates that CA IX has multiple fundamental functions in solid cancers, contemplating its pivotal role in favoring the gain of drug and radio resistance, in more advanced cases.

This finding has important diagnostic, prognostic, and therapeutic significance.

However, most of these data relies on tissue biopsies [100], which may result sub-optimal to provide detailed information about the global scenario of highly heterogeneous tumors. This could seriously hamper the monitoring of changes in clonal composition during cancer drug treatment [4].

New tumor-sampling techniques, as Circulating Tumor Cells (CTCs) collection or the analysis of circulating tumor DNA (“liquid biopsies"), could contribute to overcome this problem, considering that cancer cells are continuously shed into the circulation.

Very interestingly, Zavada *et al*. described a soluble form of CA IX, approximately 4 kDa smaller than the full-length protein comprising the extracellular domain of CA IX that is shed (sCA IX) after protolithic cleavage from the surface of tumor cells [160] in the body fluids (blood, urine) of patients with RCCC. A recent study found, in addition, soluble levels of CA IX in the urine of 70% of patients with urothelial carcinoma [161]. Zhou *et al.* reported that serum values were correlated with tumor size [162]. Recently, CA IX shedding has been reported to be a metalloprotease-dependent regulated process [163], associated with patient prognosis [53, 63, 162, 164, 165].

High serum levels of CA IX in renal, breast, cervical, and vulvar cancer correlated with circulating tumor cells (CTCs), metastasis and disease-free survival [63, 160, 164-166].

A recent study, instead, showed divergent results in ovarian tumors patients [167]. This discrepancy can be due to the restricted number of patients analyzed, or to the very effectively clearance of CA IX from the blood, operated by the kidneys [127].

The evaluation of s-CA IX by ELISA assay, in serum, reported a CA IX/s-CA IX ratio of about 10% in cancer patients, opposed to the extremely low concentration in healthy subjects [160, 166].

There is still much work to be done, to proper address the best way to assess CA IX expression in cancer tissue and body fluids. Nevertheless, CA IX appears a multi-function molecule of pathogenetic, diagnostic and therapeutic utility, representing an extraordinary example of the ideal end-product of the molecular medicine-based, post-genomic drug-diagnostic co-development model [168].

In particular, CA IX targeted drugs may be capable to block multiple pleiotropic transduction signals which strongly supports therapy resistance of hypoxic, stem-cells like cells of advanced cancer.

## Figures and Tables

**Table 1.** 

Author (Ref.)	Histotype	Method	Prognosis
SWINSON DE 2003 (55)	LUNG NSCLC	IHC - WB	POOR
KIM SJ 2005 (56)	LUNG NSCLC	IHC	POOR
ILLIE M 2010 (57)	LUNG NSCLC	IHC - ELISA	POOR
KORKEILA E 2009 (59)	RECTUM	IHC	POOR
CHIA SK 2001 (60)	BREAST	IHC	POOR
TRASTOUR C 2007 (61)	BREAST	IHC	POOR
TAN EY 2009 (62)	BREAST	IHC	POOR
LONCASTER JA 2001 (63)	UTERINE CERVIX	IHC	POOR
KIM JY 2006 (64)	UTERINE CERVIX	RT-PCR	POOR
LEE S 2007 (65)	UTERINE CERVIX	IHC	POOR
WOELBER L 2011 (66)	UTERINE CERVIX	IHC	POOR
HOSKIN PJ 2003 (67)	URINARY BLADDER	IHC	POOR
CHOSCHZICK M 2011 (68)	OVARY	IHC	POOR
NORDFORS K 2010 (69)	SNC -MEDULLOBLASTOMA – NEUROECTODERMAL TUMORS	IHC	POOR
HOOGSTEEN IJ 2005 (70)	H&N SCC	IHC	POOR
DE SCHUTTER H 2005 (71)	H&N SCC	IHC	POOR
KOUKOURAKIS MI 2006 (72)	H&N SCC	IHC	POOR
ECKERT AW 2010 (73)	OSCC	IHC	POOR
CHOI SW 2008 (74)	OSCC	IHC	POOR
HUSSAIN SA 2007 (75)	BREAST	IHC	POOR
KLATTE T 2009 (76)	URINARY BLADDER	IHC	POOR
HAAPASALO JA 2006 (77)	SNC – ASTROCYTIC TUMORS	IHC	POOR
KOUKOURAKIS MI 2001 (78)	H&N SCC	IHC	POOR
GIATROMANOLAKI A 2001 (81)	LUNG NSCLC	IHC	POOR
BRENNAN DJ (83)	BREAST	IHC - WB	POOR
PROESCHOLDT MA 2012 (84)	SNC - GLIOBLASTOMA	IHC - WB - siRNA	POOR
CLEVEN AH 2008 (111)	COLON-RECTUM	IHC	POOR
COLPAERT CG 2003 (112)	BREAST	IHC	POOR
BROCKTON N 2011 (114)	H&N SCC	quantitative fluorescent immunohistochemistry	POOR
PEREZ-SAYANS M 2012 (115)	OSCC	IHC	POOR
KIM SJ 2007 (117)	TONGUE SCC	IHC	POOR
LE QT 2007 (118)	H&N SCC	IHC	POOR
KONG CS 2009 (120)	H&N SCC	IHC	POOR
DUNGWA JV 2012 (132)	SNC - NEUROBLASTOMA	IHC	POOR
CHEN J 2005 (134)	STOMACH	WB-IHC-PCR	POOR

## ABBREVIATIONS

CA IX: = Carbonic Anhydrase IX
CSCs: = Cancer Stem Cells
CTCs: = Circulating Tumor Cells
EMT: = Epithelial Mesenchymal Transition
HIF: = Hypoxia Inducible Factor
HNSCC: = Head and Neck Squamous Cell Carcinoma
HPV: = Human Papilloma Virus
MET: = Mesenchymal Epithelial Transition
OSCC: = Oral Squamous Cell Carcinoma
PTKIs: = Protein Tyrosine Kinase Inhibitors
RCCC: = Clear Renal Cell Carcinoma
sCA IX: = Soluble Carbonic Anhydrase IX
SCC: = Squamous Cell Carcinoma
VHL tsg: = Von Hipple Lindau tumor suppressor gene
